# Impact of Antegrade Selective Cerebral Perfusion Flow Ranges on Clinical and Neurological Outcomes in Aortic Arch Surgery

**DOI:** 10.1093/icvts/ivag200

**Published:** 2026-07-15

**Authors:** Antonio Piperata, Jef Van den Eynde, Sabrina Castagnini, Fulvio Carotenuto, Riccardo Garofalo, Silvia Snaidero, Alessandro Leone, Luca Di Marco, Davide Pacini

**Affiliations:** Department of Cardiac Thoracic and Vascular Surgery, Division of Cardiac Surgery, IRCCS Azienda Ospedaliera-Universitaria di Bologna, Bologna 40138, Italy; Department of Cardiovascular Sciences, KU Leuven, Herestraat 49, box 7001, Leuven 3000, Belgium; Department of Cardiac Thoracic and Vascular Surgery, Division of Cardiac Surgery, IRCCS Azienda Ospedaliera-Universitaria di Bologna, Bologna 40138, Italy; Department of Cardiac Thoracic and Vascular Surgery, Division of Cardiac Surgery, IRCCS Azienda Ospedaliera-Universitaria di Bologna, Bologna 40138, Italy; Department of Cardiac Thoracic and Vascular Surgery, Division of Cardiac Surgery, IRCCS Azienda Ospedaliera-Universitaria di Bologna, Bologna 40138, Italy; Department of Cardiac Thoracic and Vascular Surgery, Division of Cardiac Surgery, IRCCS Azienda Ospedaliera-Universitaria di Bologna, Bologna 40138, Italy; Department of Cardiac Thoracic and Vascular Surgery, Division of Cardiac Surgery, IRCCS Azienda Ospedaliera-Universitaria di Bologna, Bologna 40138, Italy; Department of Cardiac Thoracic and Vascular Surgery, Division of Cardiac Surgery, IRCCS Azienda Ospedaliera-Universitaria di Bologna, Bologna 40138, Italy; Department of Cardiac Thoracic and Vascular Surgery, Division of Cardiac Surgery, IRCCS Azienda Ospedaliera-Universitaria di Bologna, Bologna 40138, Italy

**Keywords:** aortic arch surgery, cerebral perfusion, aortic surgery

## Abstract

**Objectives:**

To evaluate the association between selective antegrade cerebral perfusion (SACP) flow ranges and early clinical outcomes in a large cohort of patients undergoing aortic arch surgery.

**Methods:**

We retrospectively analysed 492 adult patients who underwent aortic arch surgery with bilateral antegrade cerebral perfusion under moderate hypothermia between 2015 and 2024. Patients were stratified into low, intermediate, or high-indexed SACP pump flow groups according to measured intraoperative flow rates (mL/kg/minute). Primary outcomes included in-hospital mortality, permanent neurologic dysfunction, and transient neurological deficit.

**Results:**

Overall, 80.3% of patients received antegrade selective cerebral perfusion flows within the intermediate range (10-15 mL/kg/minute). No statistically significant differences were observed among groups in terms of early mortality, permanent neurological dysfunction, or transient neurological deficit. When cerebral perfusion flow was analysed as a continuous variable, no linear association with adverse outcomes was identified (all *P* > .15). Importantly, intraoperative perfusion strategies were largely guided by clinical judgement, with flow adjustments tailored to individual patient characteristics and dynamic intraoperative factors, including extremes of body weight.

**Conclusions:**

No significant association was observed between SACP flow ranges and clinical outcomes within the flow ranges and management strategy applied in this cohort. Nonetheless, maintaining SACP within an optimal range remains advisable. Individualized flow adjustments by the clinical team, based on patient-specific factors, likely contributed to the observed flow distribution and outcomes.

## Graphical Abstract

**Figure:**
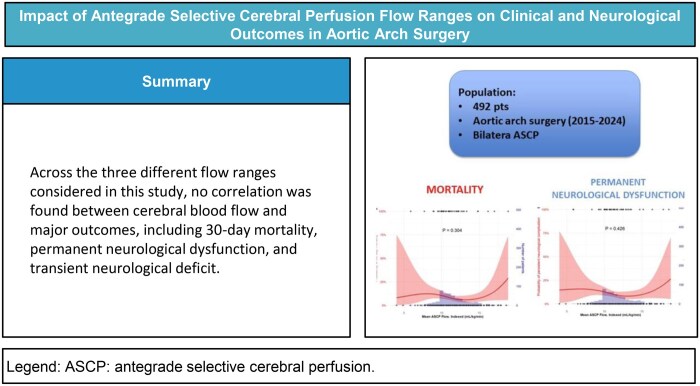

## INTRODUCTION

Cerebral blood flow (CBF) is tightly regulated through a complex interaction between perfusion pressure, flow, and metabolic demand, mediated by cerebral autoregulation mechanisms. During aortic arch surgery, these physiological processes are profoundly altered, and the balance between perfusion flow, pressure, and oxygen delivery becomes critical to maintain adequate cerebral protection.

The introduction of selective antegrade cerebral perfusion (SACP) by Kazui et al in the early 1990s marked a pivotal advancement in neuroprotection, allowing continuous CBF during circulatory arrest and significantly improving neurological outcomes.^1^^,^^2^

Selective antegrade cerebral perfusion has become the standard cerebral protection strategy, typically performed under moderate hypothermia (20-28 °C). In clinical practice, perfusion management is based on a complex interplay between flow, perfusion pressure, and cerebral oxygenation monitoring (eg, near-infrared spectroscopy, NIRS). Although weight-based formulas are commonly used to guide SACP flow, no clear consensus exists, and target flow rates generally range between 10 and 15 mL/kg/minute across centres.^3^^,^^4^

However, this method remains entirely empirical, and limited data are available regarding the optimal threshold of indexed SACP pump flow to maintain during these procedures and the impact on patients outcomes. Moreover, although simple and widely used, this model is based on the assumption that cerebral metabolic demand scales proportionally with body weight, an assumption that is physiologically debateable, particularly in patients with extreme body mass indices.

Additional variability in cerebrovascular anatomy, cannulation techniques, and institutional protocols may limit the generalizability of existing evidence and complicate the definition of a standardized cerebral perfusion strategy. In this context, the present study aims to explore the relationship between different SACP flow ranges and early clinical outcomes within a single-centre, standardized institutional protocol.

## METHODS

### Ethical statement

This study was approved by the Ethics Committee of the IRCCS University Hospital of Bologna (IRB approval number: AVECn°233/2025/Oss/AOUBo). Written informed consent was obtained from all participants prior to their inclusion in the study. Data were managed in accordance with the General Data Protection Regulation (GDPR), ensuring patient anonymity and confidentiality. All data were used exclusively for scientific purposes.

### Study population

This was a retrospective single-centre observational study including all consecutive patients undergoing aortic arch surgery between 2015 and 2024 at the IRCCS University Hospital of Bologna.

A total of 628 patients underwent aortic arch surgery during the study period. Inclusion criteria consisted of all adult patients undergoing aortic arch surgery during this period. Exclusion criteria included missing cerebral perfusion data, missing neurological outcome data, and prior history of cerebrovascular disease. After excluding 66 patients for missing cerebral perfusion data, 7 for missing neurological outcome data, and 63 with a prior history of cerebrovascular disease, 492 patients were included in the final analysis. **Figure S1** illustrates the patient selection flow chart.

### Institutional protocol for cerebral perfusion

All patients were over 18 years of age and underwent aortic arch surgery using bilateral SACP under moderate hypothermia (approximately 25 °C), with or without concomitant cardiac procedures.

The institutional protocol for cerebral perfusion has been described previously^5^ and includes, in both elective and emergency cases, bilateral antegrade cerebral perfusion through the right and left supra-aortic vessels with a target flow of 10-15 mL/kg/minute under moderate hypothermia (25 °C). This is generally achieved by establishing cardiopulmonary bypass (CPB) via the right axillary artery whenever feasible, followed by the insertion of a balloon-tipped cannula into the left carotid artery once the aortic arch is opened.

Surgical strategies, including the extent of aortic repair and cannulation techniques, were guide by the anatomical characteristics and the type and extent of the aortic disease, and were left to the discretion of the operating surgeon. In addition, specific intraoperative adjustments related to anatomical variability (eg, dominant vertebral artery or incomplete circle of Willis) were not standardized and were managed according to surgeon and perfusionist discretion.

Data were obtained from electronic medical records and clinical outcomes were defined according to international guidelines from STS and EACTS.^6^

Intraoperative cerebral monitoring routinely included NIRS and jugular venous oxygen saturation. Transcranial Doppler and continuous EEG were not systematically employed. Flow adjustments were guided by these parameters in combination with clinical judgement.

Circulatory arrest time refers to the period during which no systemic perfusion was provided to any vascular territory, including both cerebral and visceral circulation. In contrast, visceral ischaemia time refers exclusively to the duration of absent abdominal visceral perfusion and may therefore exceed circulatory arrest time when selective cerebral perfusion is employed.

### Rationale and objective of the study

In current clinical practice, SACP flow is commonly determined using weight-based formulas, typically targeting 10-15 mL/kg/minute. Although widely adopted, this approach may not fully account for inter-individual variability in cerebral perfusion requirements and often requires intraoperative adjustments.

To reflect real-world practice, patients were retrospectively categorized into 3 flow strata: low (<10 mL/kg/minute), intermediate (10-15 mL/kg/minute), and high (>15 mL/kg/minute).

Patients were categorized into 3 predefined flow strata based on institutional protocols and commonly adopted empirical targets. Although SACP flow was primarily determined using weight-based formulas, intraoperative adjustments based on monitoring and clinical judgement could result in deviations from target values.

No data-driven approaches (eg, quartiles or spline-based cut-offs) were applied.

The final goal of the study was to investigate if indexed SACP pump flow ranges influence the early clinical outcomes and if there are differences between the groups.

The clinical outcomes investigated in the study were:

- Early mortality, defined as death within 30 days after surgery.
- Permanent neurologic dysfunction (PND), defined as the presence of stroke, motor deficit (confirmed at CT/MRI control), or coma.
- Transient neurologic deficit (TND), defined as the presence of reversible postoperative deficit confusion, delirium, and motor deficits with no signs of stroke at CT scan.

### Statistical analysis

Continuous variables were reported as mean (SD) and compared between groups using analysis of variance (ANOVA), while categorical variables were reported as frequencies and percentages and compared using Chi-squared test.

Logistic regression models were fitted for the clinical outcomes (30-day mortality, PND, and TND), using restricted cubic spline-transformed SACP as a fixed effect and patient identifier as a random intercept. Restricted cubic splines were based on 3 knots according to the optimal fit method. Resulting equations from each of these models were used to calculate the predicted event probabilities and 95% CIs at the entire SACP range. Predicted event probabilities were then plotted against SACP levels to visually assess the relationships.

All analyses were completed with R Statistical Software (version 4.0.5, Foundation for Statistical Computing, Vienna, Austria).

Statistical analyses were performed to assess the association between indexed SACP flow and clinical outcomes. *P-*value refer to the relationship between SACP flow (modelled as both continuous and categorical variable) and the investigated end-points.

## RESULTS

### Study population

A total of 492 patients who underwent aortic arch surgery with SACP were included in this retrospective analysis. The mean age of the entire cohort was 64.3 years, 33.9% were females and mean EuroSCORE II was8.3%.

### SACP flow categories

**Figure 1.**
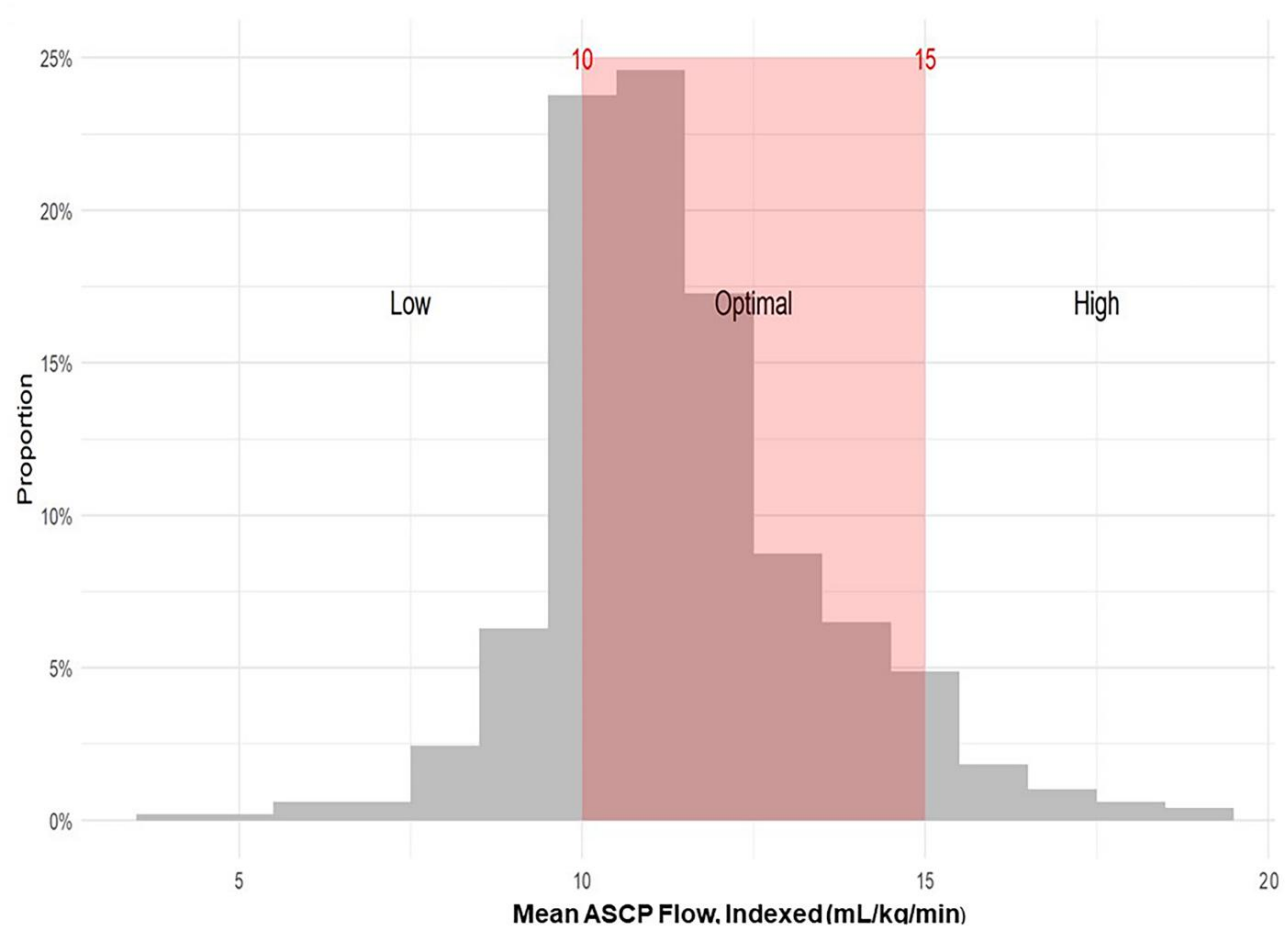
The Figure Shows the Distribution of the Sample in the 3 Different Ranges

The overall mean indexed SACP flow was 11.5 ± 2.0 mL/kg/minute and the mean absolute SACP flow was 890 ± 187 mL/minute (**Table 1**). A total of 395 patients (80.3%) were within the “intermediate” range of 10-15 mL/kg/minute, 26 patients (5.3%) received high flow (>15 mL/kg/minute), and 71 patients (14.4%) received low flow (<10 mL/kg/minute) (**Figure 1**).

**Table 1:** Pre-operative Details, Procedural Characteristics and Outcomes.

	[ALL] *N* = 492	High (>15 mL/kg/minute) *N* = 26	Low (<10 mL/kg/minute) *N* = 71	Optimal (10-15 mL/minute) *N* = 395	*P* overall
Mean absolute ASCP flow	890 ± 187	1030 ± 227	792 ± 190	899 ± 175	<.001
Mean indexed ASCP flow	11.5 ± 2.04	16.4 ± 1.10	8.72 ± 1.18	11.6 ± 1.36	<.001
Age (years)	64.3 ± 12.0	63.6 ± 14.5	60.6 ± 13.4	65.0 ± 11.4	.014
Female	167 (33.9%)	13 (50.0%)	21 (29.6%)	133 (33.7%)	.165
Weight (kg)	79.0 ± 17.2	63.0 ± 14.1	90.8 ± 18.0	78.0 ± 16.1	<.001
Height (cm)	171 ± 10.2	166 ± 9.39	176 ± 9.56	171 ± 10.1	<.001
BSA (mq)	1.93 ± 0.25	1.70 ± 0.23	2.09 ± 0.24	1.91 ± 0.23	<.001
BMI	26.8 ± 4.82	22.5 ± 3.64	29.3 ± 4.79	26.7 ± 4.66	<.001
EuroSCOREII (%)	8.26 ± 5.80	10.0 ± 6.53	7.03 ± 3.68	8.37 ± 6.04	.061
LVEF (%)	59.7 ± 6.74	58.6 ± 10.7	59.3 ± 7.57	59.9 ± 6.24	.546
Preoperative renal failure, *n* (%)	42 (8.59%)	1 (3.85%)	5 (7.14%)	36 (9.16%)	.773
Diabetes, *n* (%)	32 (6.53%)	1 (3.85%)	5 (7.04%)	26 (6.62%)	.937
Smoking, *n* (%)	190 (38.7%)	10 (38.5%)	27 (38.0%)	153 (38.8%)	.992
COPD, *n* (%)	2 (3.64%)	0 (0.00%)	1 (11.1%)	1 (2.22%)	.333
TIA, *n* (%)	491 (100%)	26 (100%)	71 (100%)	394 (100%)	.
Preoperative stroke, *n* (%)	492 (100%)	26 (100%)	71 (100%)	395 (100%)	.
Marfan syndrome, *n* (%)	13 (2.65%)	0 (0.00%)	3 (4.23%)	10 (2.54%)	.506
LoeysDietz syndrome, *n* (%)	1 (0.20%)	1 (3.85%)	0 (0.00%)	0 (0.00%)	.053
Redo surgery, *n* (%)	134 (27.3%)	14 (53.8%)	14 (20.0%)	106 (26.9%)	.004
Urgency					.004
Elective, *n* (%)	206 (41.9%)	16 (61.5%)	19 (26.8%)	171 (43.3%)	
Urgency/emergency, *n* (%)	286 (58.1%)	10 (38.5%)	52 (73.2%)	224 (56.7%)	
Type B dissection, *n* (%)	29 (5.89%)	2 (7.69%)	2 (2.82%)	25 (6.33%)	.442
Type A dissection, *n* (%)	232 (47.2%)	6 (23.1%)	48 (67.6%)	178 (45.1%)	<.001
Aneurysm, *n* (%)	182 (37.0%)	15 (57.7%)	19 (26.8%)	148 (37.5%)	.018
Replacement extension, *n* (%)					>.05
Elephant trunk, *n* (%)	22 (4.47%)	1 (3.85%)	3 (4.23%)	18 (4.56%)	
Frozen elephant trunk, *n* (%)	179 (36.4%)	12 (46.2%)	25 (35.2%)	142 (35.9%)	
Hemiarch replacement, *n* (%)	183 (37.2%)	8 (30.8%)	26 (36.6%)	149 (37.7%)	
Other, *n* (%)	5 (1.02%)	0 (0.00%)	2 (2.82%)	3 (0.76%)	
Partial/total arch, *n* (%)	103 (20.9%)	5 (19.2%)	15 (21.1%)	83 (21.0%)	
Cannulation type					>.05
Arch, *n* (%)	16 (3.25%)	1 (3.85%)	2 (2.82%)	13 (3.29%)	
Ascending aorta, *n* (%)	52 (10.6%)	1 (3.85%)	5 (7.04%)	46 (11.6%)	
Axillary, *n* (%)	80 (16.3%)	4 (15.4%)	12 (16.9%)	64 (16.2%)	
Axillary and carotid, *n* (%)	1 (0.20%)	0 (0.00%)	0 (0.00%)	1 (0.25%)	
Brachiocephalic trunk, *n* (%)	141 (28.7%)	16 (61.5%)	15 (21.1%)	110 (27.8%)	
Carotid artery, *n* (%)	43 (8.74%)	1 (3.85%)	3 (4.23%)	39 (9.87%)	
Femoral artery, *n* (%)	159 (32.3%)	3 (11.5%)	34 (47.9%)	122 (30.9%)	
Concomitant CABG, *n* (%)	31 (6.30%)	0 (0.00%)	5 (7.04%)	26 (6.58%)	.535
Concomitant AVR, *n* (%)	22 (4.47%)	1 (3.85%)	1 (1.41%)	20 (5.06%)	.411
Concomitant_Bentall, *n* (%)	175 (35.6%)	8 (30.8%)	24 (33.8%)	43 (36.2%)	.881
CPB time (minutes)	221 ± 65.3	208 ± 71.8	240 ± 81.5	219 ± 61.1	.025
Aortic Xclamp time (minutes)	138 ± 50.0	126 ± 55.6	143 ± 54.6	138 ± 48.7	.337
Circulatory arrest time (minutes)	3.87 ± 12.8	1.92 ± 2.19	5.21 ± 15.5	3.76 ± 12.7	.496
Time of ASCP (minutes)	74.9 ± 45.6	73.3 ± 27.4	87.8 ± 60.3	72.7 ± 43.1	.036
Time of_Visceral ischaemia (minutes)	40.5 ± 15.6	42.0 ± 15.9	41.9 ± 16.6	40.2 ± 15.4	.610
Nasopharingeal Temp (minutes)	25.0 ± 1.03	24.8 ± 1.59	24.9 ± 0.86	25.0 ± 1.01	.646
ICU_stay (days)	11.3 ± 19.2	7.81 ± 12.6	12.1 ± 20.4	11.4 ± 19.3	.614
Hospital_stay (days)	24.8 ± 24.0	17.1 ± 12.9	25.4 ± 26.7	25.2 ± 24.0	.243
Intubation longer than 72 hours, *n* (%)	136 (28.1%)	6 (23.1%)	23 (32.9%)	107 (27.6%)	.560
PND, *n* (%)	54 (11.0%)	4 (15.4%)	6 (8.45%)	44 (11.2%)	.582
Stroke, *n* (%)	40 (8.13%)	4 (15.4%)	5 (7.04%)	31 (7.85%)	.390
Paraplegia, *n* (%)	15 (3.05%)	0 (0.00%)	2 (2.82%)	13 (3.29%)	1.000

Circulatory arrest time refers to the period during which no systemic perfusion was provided to any vascular territory, including both cerebral and visceral circulation. In contrast, visceral ischaemia time refers exclusively to the duration of absent abdominal visceral perfusion and may therefore exceed circulatory arrest time when selective cerebral perfusion is employed.

Abbreviations: ASCP, antegrade selective cerebral perfusion; AVR, aortic valve replacement; BCT, brachiocephalic trunk; BMI, body mass index; BSA, body surface area; CABG, coronary artery bypass grafting; CPB, cardiopulmonary bypass; COPD, chronic obstructive pulmonary disease; ICU, intensive care unit; LVEF, left ventricular ejection fraction; PND, permanent neurological dysfunction.

### Relationship of SACP flow with clinical variables

As shown in **Table 1**, patients in the low flow group were significantly younger than those in the intermediate group, but had higher weight, height, BSA, and BMI (mean weight 90.8 ± 18 kg) compared to both the intermediate and high flow groups. A low indexed flow strategy was preferentially adopted in urgent/emergency surgeries (73.2%), acute type A aortic dissections (67.6%), and in patients with longer CPB durations (240 ± 81.5 minutes) and longer SACP times (87.8 ± 60.3 minutes).

In contrast, high flow was significantly more often employed in patients undergoing redo surgeries (53.8%), those with prior vascular interventions (30.8%), and in the presence of chronic aortic aneurysms (57.7%).

### Relationship of SACP flow with outcomes

**Figure 2.**
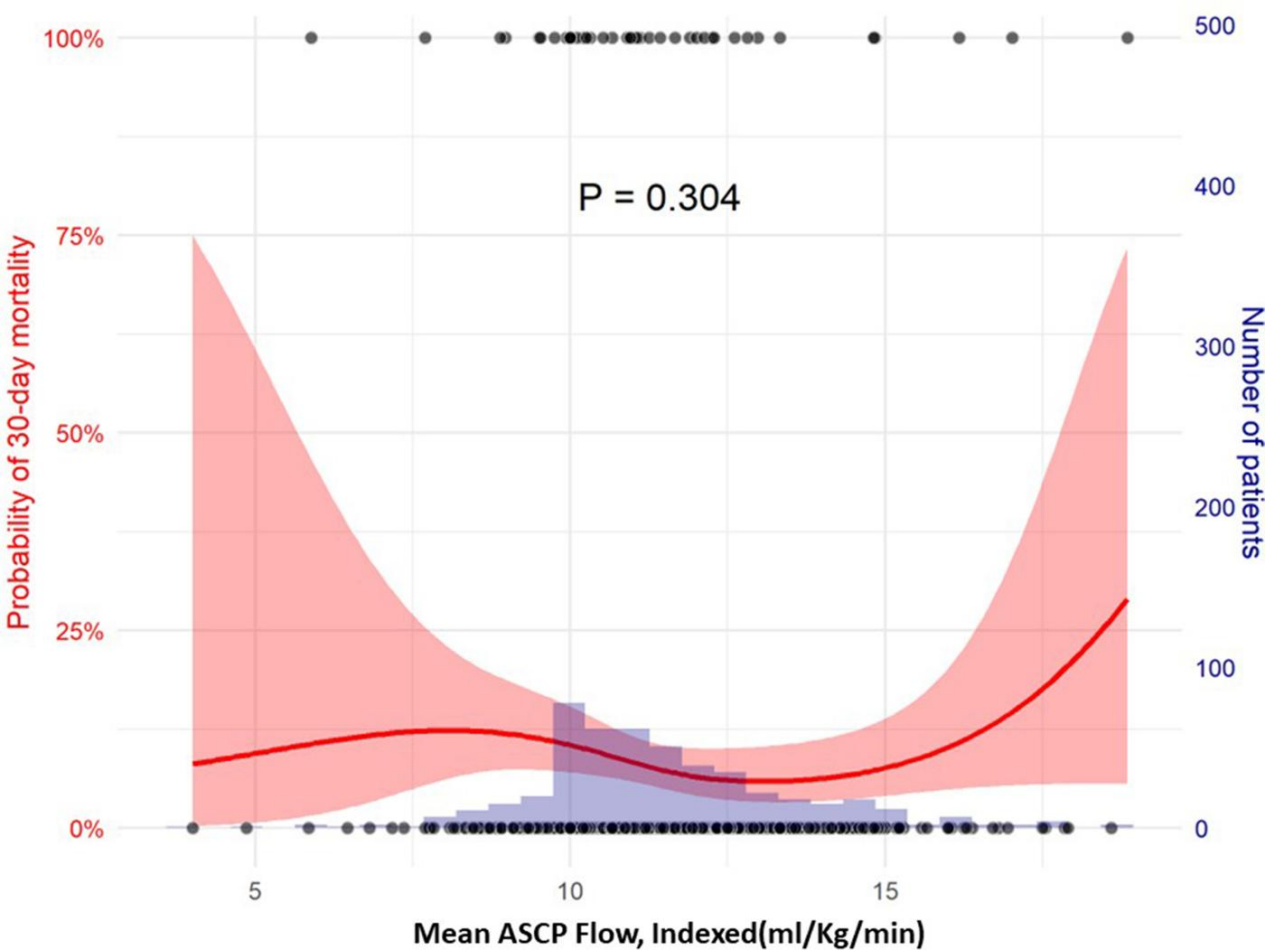
Relationship Between Indexed SACP Flow and the Probability of 30-Day Mortality. The red solid line represents the estimated probability derived from spline regression analysis, while the shaded area indicates the 95% CI. Black dots represent individual patient data points. The blue histogram reflects the distribution of patients across the range of SACP flow values. The *P*-value refers to the overall association between SACP flow (modelled as a continuous variable) and the outcome

**Figure 3.**
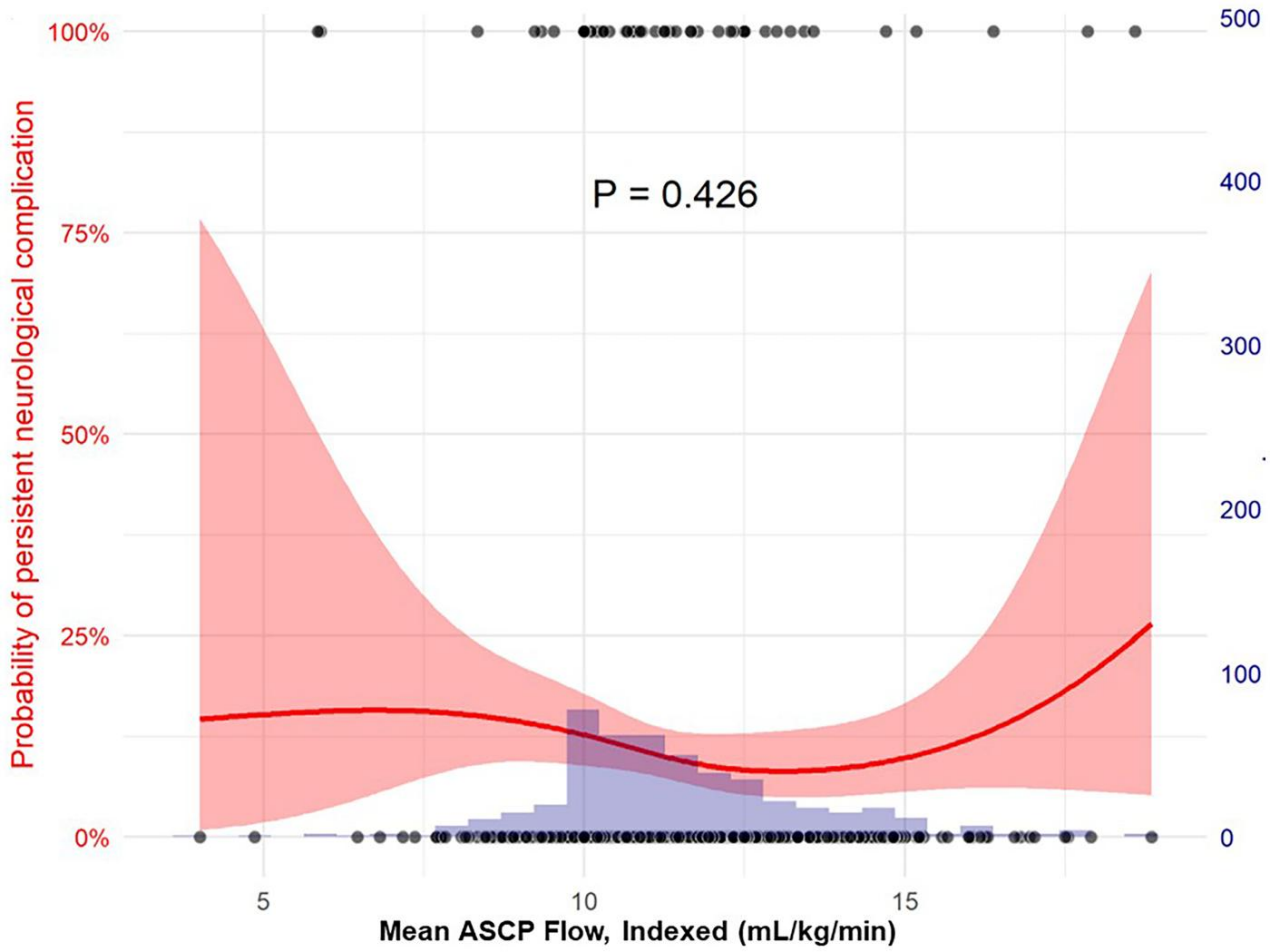
Relationship Between Indexed SACP Flow and the Probability of Permanent Neurological Dysfunction. The red solid line represents the estimated probability derived from spline regression analysis, while the shaded area indicates the 95% CI. Black dots represent individual patient data points. The blue histogram reflects the distribution of patients across the range of SACP flow values. The *P*-value refers to the overall association between SACP flow (modelled as a continuous variable) and the outcome

**Figure 4.**
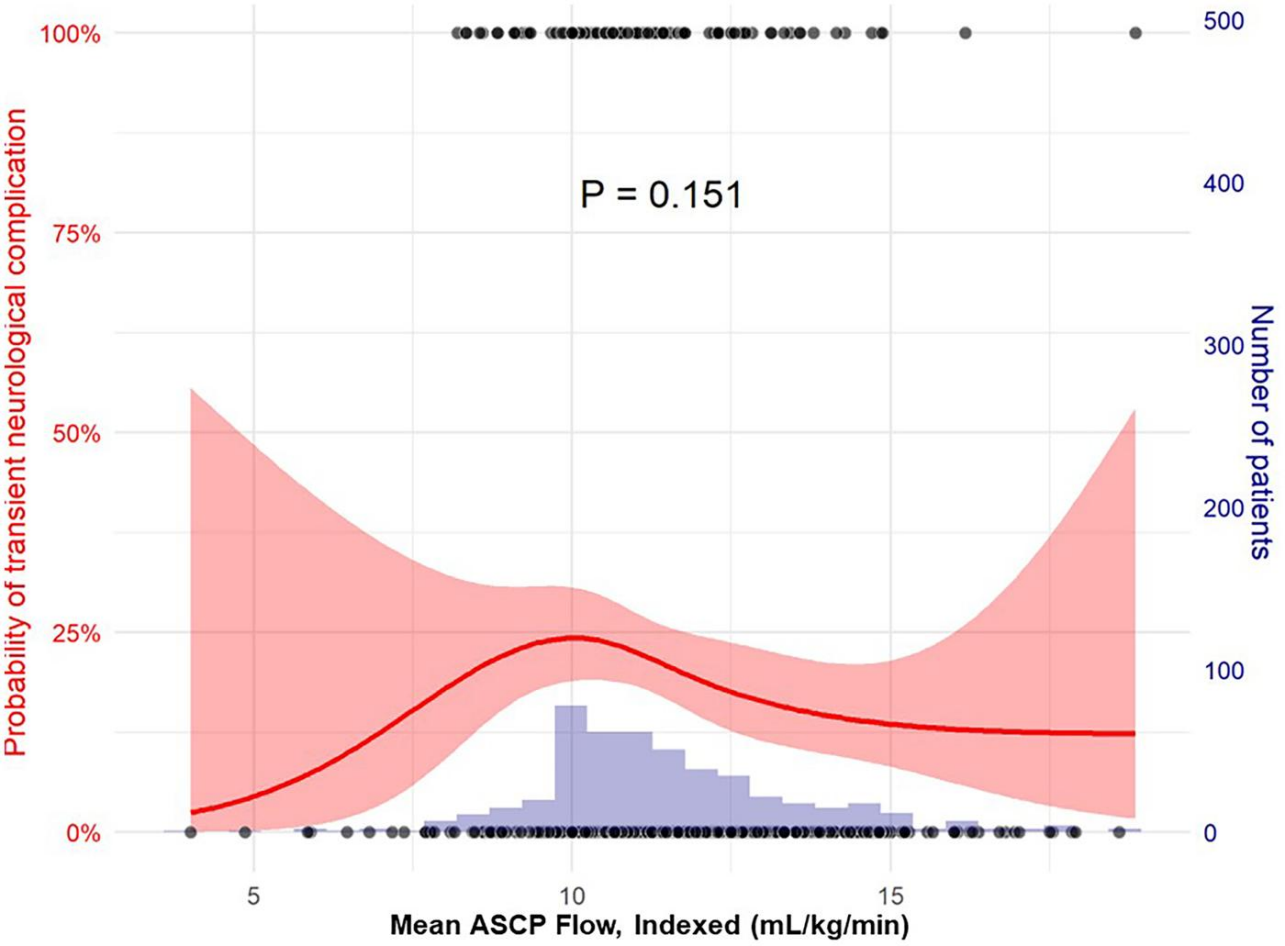
Relationship Between Indexed SACP Flow and the Probability of Transient Neurological Deficit. The red solid line represents the estimated probability derived from spline regression analysis, while the shaded area indicates the 95% CI. Black dots represent individual patient data points. The blue histogram reflects the distribution of patients across the range of SACP flow values. The *P*-value refers to the overall association between SACP flow (modelled as a continuous variable) and the outcome

**Figures 2–4** illustrate the probability of 30-day mortality, PND, and TND, respectively, as a function of SACP flow. Across indexed SACP flow values, the rates of adverse outcomes were comparable. The statistical analyses (all *P-*value > .15) suggest that variations in flow within the observed ranges did not significantly influence the studied outcomes.

In addition, no statistically significant differences were observed when outcomes were analysed dichotomously, comparing patients with and without early mortality, PND, or TND (**Tables S1–S3**, respectively).

To account for potential ambiguity in the definition of the “intermediate” indexed perfusion group originally classified within a relatively broad range (10-15 mL/kg/minute) we performed a sensitivity analysis by redefining the high-indexed group as patients receiving ≥12.5 mL/kg/minute. This was done to test the robustness of our findings given the lack of universally established thresholds. The results remained consistent, confirming no significant differences in clinical outcomes (see **Table S4**).

## DISCUSSION

In this study, no statistically significant association was observed between indexed SACP flow ranges and adverse outcomes within the management strategy applied in this cohort. The most appropriate conclusion for this research is that, within a surgical program paying close attention to SACP flow recommendations during aortic arch surgery, deviations are rare and do not seem to be associated with major increases in adverse outcomes. However, as previously emphasized, although the institutional practice is to perform SACP using a weight-indexed strategy with an optimal target range of approximately 10-15 mL/kg/minute, perfusion flow rates are, of course, adjusted in real time according to the patient’s specific conditions, including haemodynamic, metabolic, and anatomical factors. This continuous adjustment is an integral and essential part of our methodological approach in this type of procedure and may have inevitably influenced the results presented. Moreover, the higher prevalence of emergency cases and acute aortic dissections in the low-flow group may have influenced outcomes. At the same time, there is a substantial proportion of patients underwent hemiarch procedures with relatively short circulatory arrest times, which may have limited the ability to detect differences related to cerebral perfusion strategies. Although statistical adjustment was performed, residual confounding cannot be excluded.

These findings likely reflect a well-standardized institutional protocol, with most patients treated within a relatively narrow flow range. Therefore, the results should not be interpreted as evidence of the irrelevance of flow, but rather as an absence of detectable differences within the applied strategy.

For all these reasons, the study was not sufficiently powered to test the effect of deviations at the extremes of the spectrum; therefore, it remains advisable to maintain SACP flow within the defined “optimal/intermediate” windows as much as possible.

Cerebral protection during aortic arch surgery remains a complex challenge due to the multitude of variables involved, including perfusion pressure, flow rates, temperature, pH, haematocrit, and cannulation sites. The individual contribution of each of these factors to clinical outcomes is difficult to quantify with precision.^7^ In addition, during CPB, the interaction between flow dynamics, other CPB parameters, and patient-specific physiological factors limits the ability to accurately predict the most appropriate flow rate for each clinical scenario. Previous studies have suggested that a flow rate exceeding 10 mL/kg/minute may be necessary when SACP is employed, as indexed SACP pump flow was found to be significantly lower in patients who experienced adverse neurological events.^8^ However, these findings are derived from highly specific surgical settings, where parameters such as temperature, pressure, and flow are not easily comparable across studies. This variability likely contributes to the inconsistency in outcomes reported in the literature. While some investigations have demonstrated signs of cerebral distress, such as increased cerebral lactate and decreased venous oxygen saturation when flow rates drop below 6 mL/kg/minute,^9^ other clinical reports have documented the use of flow rates below 5 mL/kg/minute without any apparent increase in neurological complications.^10–12^ These divergent findings underscore the difficulty of establishing universally applicable flow thresholds, as outcomes are heavily influenced by the specific perfusion strategies and intraoperative conditions employed.

Nevertheless, in the absence of a clear consensus, SACP flow rates in clinical practice vary widely across centres, typically ranging from 8.5 to14.3 mL/kg/minute in earlier series,^9^ and from 10 to 15 mL/kg/minute in more recent reports.^1^^,^^2^ The findings of the present study contribute to the ongoing debate regarding the complexity of cerebral circulation and highlights that outcomes in patients undergoing SACP are surely multifactorial.

The adult human brain is a unique organ. Although it comprises only 2%-3%of total body mass, it receives approximately 15% of the cardiac output and consumes around 20% of the body’s available oxygen under normal conditions.^13^ To meet these high metabolic demands, CBF plays a vital role, underscored by the severe and lasting damage that follows significant reductions in CBF.^14^^,^^15^ In awake individuals, normal CBF is roughly 50 mL/100 g/minute, with functional impairment typically observed when levels fall below approximately 22 mL/100 g/minute.^14^ While no absolute thresholds for ischaemic injury have been defined, it is generally estimated that a sustained reduction to 20%-30% of baseline CBF can induce ischaemia within minutes.^15–17^ However, this estimate may overstate the risk, as ischaemic vulnerability varies across brain cell populations and between core and penumbra regions during stroke. Moreover, in addition to the immediate effects of hypoperfusion, a cascade of secondary responses can be triggered, leading to progressive damage and delayed ischaemia over the following hours or even days.^15^

Cardiopulmonary bypass is maintained through 4 primary adaptive mechanisms: cerebral perfusion pressure, mainly influenced by arterial blood pressure^18–24^; chemoregulation, reflecting vascular responses to changes in CO_2_ and O_2_ levels^25–27^; neural activity; and the modulatory role of vascular endothelial cells. Increasing evidence shows that these mechanisms are not isolated but operate in a tightly integrated manner to preserve brain health.^18^^,^^19^ The complex and dynamic interaction among these regulatory systems is specific to the brain, helping explain why peripheral vascular models poorly predict cerebral vascular behaviour.^28^ For all these reasons, no robust conclusions can be drawn from the currently available evidence. Ultimately, it must be acknowledged that current CBF models have important limitations, and neither represents a perfect solution. At present, these approaches remain largely empirical, and we are likely still far from a truly individualized method for determining the optimal CBF in these patients. This stems from the fact that numerous unquantifiable and often undetected factors such as anatomical variations, unrecognized cerebrovascular pathologies, auto-regulatory mechanisms, and temperature effects play significant roles that we are not yet able to accurately measure or respond to in real time.

In conclusion, no significant association was observed between SACP flow ranges and clinical outcomes within the applied institutional protocol.

In the absence of clear consensus and high-quality evidence supporting a universal optimal flow threshold, a rigid flow-based strategy may be inherently limited. Rather, the data support a patient-tailored approach, in which cerebral perfusion is continuously adapted to individual physiological conditions. Such an individualized strategy should account for dynamic intraoperative changes in metabolic demand, temperature, haemodynamics, and cerebrovascular autoregulation, allowing real-time modulation of flow to ensure adequate cerebral oxygen delivery while avoiding excessive perfusion and its potential complications.

There are several limitations to this study that warrant consideration. First, its retrospective design inherently limits both the completeness and granularity of the collected data, while although the study was conducted in a high-volume aortic surgery centre, the sample size remains insufficient to allow for robust subgroup analyses. Second, the inclusion of patients with shorter circulatory arrest times, particularly in hemiarch procedures, may have diluted potential differences between groups, and, heterogeneity in case mix, including a higher proportion of acute dissections and reoperations in specific groups, may have influenced outcomes.

Third, indexed pump flow was also used as a surrogate of cerebral perfusion; however, it does not directly reflect CBF, and the absence of systematic use of advanced monitoring tools such as transcranial Doppler or EEG represents another important limitation. In addition, the unbalanced distribution among flow groups, with a predominance of patients in the intermediate range, reflects real-world practice but may have limited the statistical power to detect differences, particularly in the extreme groups.

Nonetheless, our findings lay the groundwork for a prospective, randomized trial aimed at establishing more precise and patient-tailored criteria for SACP in aortic arch surgery.

## Supplementary Material

ivag200_Supplementary_Data

## Data Availability

The data cannot be shared for privacy reasons.

## ABBREVIATIONS

AATS: American Association of Thoracic Surgery
BMI: body mass index
BSA: body surface area
CABG: coronary artery bypass graft
CBF: cerebral blood flow
EACTS: European Association of Cardio-Thoracic Surgery
PND: permanent neurological dysfunction
SACP: selective antegrade cerebral perfusion
STS: Society of Thoracic Surgery
TND: transient neurological deficit
