# Supplementary material for: Impact of Antegrade Selective Cerebral Perfusion Flow Ranges on Clinical and Neurological Outcomes in Aortic Arch Surgery
Source: Interdiscip Cardiovasc Thorac Surg. 2026 Jul 15;41(8):ivag200. doi: 10.1093/icvts/ivag200 (PMC13431124; doi:10.1093/icvts/ivag200)
Supplement: ivag200_Supplementary_Data [file ivag200_supplementary_data.zip › TABLE 4 SUPPLEMENTARY.docx]

Supplementary table S4

|  | [ALL] N=492 | <12.5 mL/kg/min N=363 | >12.5 mL/kg/min N=129 | p.overall |
| --- | --- | --- | --- | --- |
| Mean ASCP Flow absolute (ml/min) | 890 (187) | 864 (172) | 963 (206) | <0.001 |
| MeanASCP flow Indexed (ml/kg/min) | 11.5 (2.04) | 10.5 (1.23) | 14.1 (1.45) | <0.001 |
| Age (y) | 64.3 (12.0) | 63.5 (12.0) | 66.5 (11.8) | 0.015 |
| Female | 167 (33.9%) | 116 (32.0%) | 51 (39.5%) | 0.146 |
| Weight(kg) | 79.0 (17.2) | 82.8 (16.7) | 68.5 (14.3) | <0.001 |
| Height(cm) | 171 (10.2) | 172 (10.3) | 168 (9.44) | <0.001 |
| BSA(mq) | 1.93 (0.25) | 1.98 (0.24) | 1.78 (0.22) | <0.001 |
| BMI | 26.8 (4.82) | 27.8 (4.74) | 24.0 (3.80) | <0.001 |
| EuroSCOREII(%) | 8.26 (5.80) | 8.12 (6.08) | 8.65 (4.97) | 0.347 |
| LVEF(%) | 59.7 (6.74) | 59.8 (6.48) | 59.6 (7.43) | 0.826 |
| Preoperative Renal Failure n(%) | 42 (8.59%) | 31 (8.61%) | 11 (8.53%) | 1.000 |
| Diabetes n(%) | 32 (6.53%) | 26 (7.20%) | 6 (4.65%) | 0.424 |
| Smoking n(%) | 190 (38.7%) | 141 (39.0%) | 49 (38.0%) | 0.930 |
| COPD n(%) | 2 (3.64%) | 2 (4.35%) | 0 (0.00%) | 1.000 |
| TIA n(%) | 491 (100%) | 362 (100%) | 129 (100%) | . |
| Preoperative Stroke n(%) | 492 (100%) | 363 (100%) | 129 (100%) | . |
| Marfan n(%) | 13 (2.65%) | 12 (3.32%) | 1 (0.78%) | 0.200 |
| Loeys Dietz n(%) | 1 (0.20%) | 0 (0.00%) | 1 (0.78%) | 0.263 |
| REDO SURGERY | 134 (27.3%) | 86 (23.8%) | 48 (37.5%) | 0.004 |
| Urgency: |  |  |  | <0.001 |
| Elective | 206 (41.9%) | 131 (36.1%) | 75 (58.1%) |  |
| Urgency/Emergency | 286 (58.1%) | 232 (63.9%) | 54 (41.9%) |  |
| Type B Dissection n(%) | 29 (5.89%) | 24 (6.61%) | 5 (3.88%) | 0.360 |
| Type A Dissection n(%) | 232 (47.2%) | 194 (53.4%) | 38 (29.5%) | <0.001 |
| Aneurysm n(%) | 182 (37.0%) | 114 (31.4%) | 68 (52.7%) | <0.001 |
| Replacement Extension n(%) |  |  |  | 0.002 |
| Elephant Trunk | 22 (4.47%) | 17 (4.68%) | 5 (3.88%) |  |
| Frozen Elephant Trunk | 179 (36.4%) | 113 (31.1%) | 66 (51.2%) |  |
| Hemiarch | 183 (37.2%) | 148 (40.8%) | 35 (27.1%) |  |
| Other | 5 (1.02%) | 4 (1.10%) | 1 (0.78%) |  |
| Partial/Total Arch | 103 (20.9%) | 81 (22.3%) | 22 (17.1%) |  |
| Cannulation Type n(%) |  |  |  | . |
| Arch | 16 (3.25%) | 15 (4.13%) | 1 (0.78%) |  |
| Ascending Aorta | 52 (10.6%) | 41 (11.3%) | 11 (8.53%) |  |
| Axillary | 80 (16.3%) | 59 (16.3%) | 21 (16.3%) |  |
| Axillary+Carotid | 1 (0.20%) | 1 (0.28%) | 0 (0.00%) |  |
| BCT | 141 (28.7%) | 84 (23.1%) | 57 (44.2%) |  |
| Carotid | 43 (8.74%) | 23 (6.34%) | 20 (15.5%) |  |
| Femoral | 159 (32.3%) | 140 (38.6%) | 19 (14.7%) |  |
| Concomitant CABG n(%) | 31 (6.30%) | 26 (7.16%) | 5 (3.88%) | 0.268 |
| Concomitant AVR n(%) | 22 (4.47%) | 11 (3.03%) | 11 (8.53%) | 0.019 |
| Concomitant Bentall n(%) | 175 (35.6%) | 135 (37.2%) | 40 (31.0%) | 0.438 |
| CPB Time (min) | 221 (65.3) | 226 (65.2) | 208 (64.4) | 0.011 |
| Aortic clamp time (min) | 138 (50.0) | 139 (48.8) | 134 (53.3) | 0.336 |
| Circulatory arrest time (min) | 3.87 (12.8) | 4.29 (13.6) | 2.68 (10.5) | 0.168 |
| Time of ASCP (min) | 74.9 (45.6) | 75.0 (48.4) | 74.8 (36.8) | 0.965 |
| Time of Visceral Ischemia (min) | 40.5 (15.6) | 40.7 (15.8) | 40.2 (15.0) | 0.774 |
| Nasopharingeal Temp (°C) | 25.0 (1.03) | 25.0 (1.04) | 25.0 (0.99) | 0.992 |
| ICU stay (days) | 11.3 (19.2) | 11.6 (18.5) | 10.5 (21.0) | 0.619 |
| Hospital stay (days) | 24.8 (24.0) | 25.1 (23.8) | 23.8 (24.7) | 0.589 |
| Intubation Longer than 72 hours n(%) | 136 (28.1%) | 106 (29.9%) | 30 (23.3%) | 0.189 |
| Complications PND n(%) | 54 (11.0%) | 41 (11.3%) | 13 (10.1%) | 0.822 |
| Complications Stroke n(%) | 40 (8.13%) | 30 (8.26%) | 10 (7.75%) | 1.000 |
| Paraplegia | 15 (3.05%) | 12 (3.31%) | 3 (2.33%) | 0.769 |
