# Supplementary material for: Impact of Antegrade Selective Cerebral Perfusion Flow Ranges on Clinical and Neurological Outcomes in Aortic Arch Surgery
Source: Interdiscip Cardiovasc Thorac Surg. 2026 Jul 15;41(8):ivag200. doi: 10.1093/icvts/ivag200 (PMC13431124; doi:10.1093/icvts/ivag200)
Supplement: ivag200_Supplementary_Data [file ivag200_supplementary_data.zip › TABLE 1 SUPPLEMENTARY.docx]

TABLE 1 SUPPLEMENTARY

|  | Overall  n=492 | Patients Alive at 30 Days n=449 | Patients Deceased at 30 Days  n=43 | p.overall |
| --- | --- | --- | --- | --- |
| ASCP_Category_Indexed: |  |  |  | 0.480 |
| High (>15 mL/kg/min) | 26 (5.28%) | 23 (5.12%) | 3 (6.98%) |  |
| Low (<10 mL/kg/min) | 71 (14.4%) | 63 (14.0%) | 8 (18.6%) |  |
| Optimal (10-15 mL/min) | 395 (80.3%) | 363 (80.8%) | 32 (74.4%) |  |
| Mean ASCP Flow absolute | 890 (187) | 891 (185) | 878 (205) | 0.690 |
| MeanASCP flow Indexed | 11.5 (2.04) | 11.5 (2.01) | 11.3 (2.36) | 0.675 |
| Age (y) | 64.3 (12.0) | 64.0 (12.1) | 67.9 (9.65) | 0.017 |
| Female | 167 (33.9%) | 148 (33.0%) | 19 (44.2%) | 0.188 |
| Weight (kg) | 79.0 (17.2) | 79.0 (17.0) | 79.4 (19.7) | 0.898 |
| Height (cm) | 171 (10.2) | 171 (10.3) | 170 (8.49) | 0.297 |
| BSA (mq) | 1.93 (0.25) | 1.93 (0.25) | 1.92 (0.25) | 0.757 |
| BMI | 26.8 (4.82) | 26.8 (4.66) | 27.4 (6.26) | 0.515 |
| EuroSCOREII (%) | 8.26 (5.80) | 7.77 (4.31) | 13.4 (12.9) | 0.009 |
| LVEF(%) | 59.7 (6.74) | 59.8 (6.52) | 58.7 (8.75) | 0.442 |
| Preoperative Renal Failure n(%) | 42 (8.59%) | 39 (8.74%) | 3 (6.98%) | 1.000 |
| Diabetes n(%) | 32 (6.53%) | 30 (6.71%) | 2 (4.65%) | 1.000 |
| Smoking n(%) | 190 (38.7%) | 170 (37.9%) | 20 (46.5%) | 0.348 |
| COPD n(%) | 2 (3.64%) | 1 (2.00%) | 1 (20.0%) | 0.175 |
| TIA n(%) | 491 (100%) | 448 (100%) | 43 (100%) | . |
| Preoperative Stroke n(%) | 492 (100%) | 449 (100%) | 43 (100%) | . |
| Marfan n(%) | 13 (2.65%) | 13 (2.91%) | 0 (0.00%) | 0.617 |
| Loeys Dietz n(%) | 1 (0.20%) | 1 (0.22%) | 0 (0.00%) | 1.000 |
| REDO SURGERY n(%) | 134 (27.3%) | 124 (27.7%) | 10 (23.8%) | 0.721 |
| Urgency: |  |  |  | 0.001 |
| Elective | 206 (41.9%) | 199 (44.3%) | 7 (16.3%) |  |
| Urgency/Emergency | 286 (58.1%) | 250 (55.7%) | 36 (83.7%) |  |
| Type B Dissection n(%) | 29 (5.89%) | 27 (6.01%) | 2 (4.65%) | 1.000 |
| Type A Dissection n(%) | 232 (47.2%) | 203 (45.2%) | 29 (67.4%) | 0.009 |
| Aneurysm n(%) | 182 (37.0%) | 174 (38.8%) | 8 (18.6%) | 0.014 |
| Replacement Extension n(%) |  |  |  | 0.902 |
| Elephant Trunk | 22 (4.47%) | 20 (4.45%) | 2 (4.65%) |  |
| Frozen Elephant Trunk | 179 (36.4%) | 163 (36.3%) | 16 (37.2%) |  |
| Hemiarch | 183 (37.2%) | 165 (36.7%) | 18 (41.9%) |  |
| Other | 5 (1.02%) | 5 (1.11%) | 0 (0.00%) |  |
| Partial/Total Arch | 103 (20.9%) | 96 (21.4%) | 7 (16.3%) |  |
| Cannulation Type n(%) |  |  |  | 0.797 |
| Arch | 16 (3.25%) | 15 (3.34%) | 1 (2.33%) |  |
| AscendingAorta | 52 (10.6%) | 47 (10.5%) | 5 (11.6%) |  |
| Axillary | 80 (16.3%) | 75 (16.7%) | 5 (11.6%) |  |
| Axillary+Carotid | 1 (0.20%) | 1 (0.22%) | 0 (0.00%) |  |
| BCT | 141 (28.7%) | 131 (29.2%) | 10 (23.3%) |  |
| Carotid | 43 (8.74%) | 38 (8.46%) | 5 (11.6%) |  |
| Femoral | 159 (32.3%) | 142 (31.6%) | 17 (39.5%) |  |
| Concomitant CABG n(%) | 31 (6.30%) | 23 (5.12%) | 8 (18.6%) | 0.003 |
| Concomitant AVR n(%) | 22 (4.47%) | 21 (4.68%) | 1 (2.33%) | 0.710 |
| Concomitant Bentall n(%) | 175 (35.6%) | 154 (34.3%) | 21 (48.8%) | 0.008 |
| CPB Time (min) | 221 (65.3) | 215 (55.7) | 282 (112) | <0.001 |
| Aortic clamp time (min) | 138 (50.0) | 136 (48.6) | 160 (59.2) | 0.014 |
| Circulatory arrest time (min) | 3.87 (12.8) | 3.96 (13.4) | 2.95 (4.52) | 0.285 |
| Time of ASCP (min) | 74.9 (45.6) | 74.5 (45.2) | 79.5 (49.7) | 0.530 |
| Time of Visceral Ischemia (min) | 40.5 (15.6) | 40.3 (15.7) | 43.2 (14.4) | 0.219 |
| Nasopharingeal Temperature | 25.0 (1.03) | 25.0 (0.96) | 24.9 (1.57) | 0.729 |
| ICU stay (days) | 11.3 (19.2) | 11.8 (19.9) | 6.07 (7.33) | <0.001 |
| Hospital stay (days) | 24.8 (24.0) | 26.5 (24.3) | 6.63 (7.45) | <0.001 |
| Intubation Longer than 72 hours n(%) | 136 (28.1%) | 112 (25.3%) | 24 (57.1%) | <0.001 |
| Complications PND n(%) | 54 (11.0%) | 44 (9.82%) | 10 (23.3%) | 0.017 |
| ComplicationsStroke n(%) | 40 (8.13%) | 33 (7.35%) | 7 (16.3%) | 0.070 |

ASCP: antegrade selective cerebral perfusion, AVR: aortic valve replacement; BCT: brachiocephalic trunk; BMI: body mass index; BSA: body surface area; CABG: coronary artery bypass grafting; CPB: cardiopulmonary bypass; COPD: chronic obstructive pulmonary disease; ICU: intensive care unit; LVEF: left ventricular ejection fraction; PND: permanent neurological dysfunction.
