# Supplementary material for: Impact of Antegrade Selective Cerebral Perfusion Flow Ranges on Clinical and Neurological Outcomes in Aortic Arch Surgery
Source: Interdiscip Cardiovasc Thorac Surg. 2026 Jul 15;41(8):ivag200. doi: 10.1093/icvts/ivag200 (PMC13431124; doi:10.1093/icvts/ivag200)
Supplement: ivag200_Supplementary_Data [file ivag200_supplementary_data.zip › TABLE 2 SUPPLEMENTARY.docx]

TABLE 2 SUPPLEMENTARY

|  | Overall N=491 | Absence of permanent neurological dysfunction N=437 | Permanent neurological dysfunction  N=54 | p.overall |
| --- | --- | --- | --- | --- |
| ASCP_Category_Indexed: |  |  |  | 0.582 |
| High (>15 mL/kg/min) | 26 (5.30%) | 22 (5.03%) | 4 (7.41%) |  |
| Low (<10 mL/kg/min) | 71 (14.5%) | 65 (14.9%) | 6 (11.1%) |  |
| Optimal (10-15 mL/min) | 394 (80.2%) | 350 (80.1%) | 44 (81.5%) |  |
| Mean ASCP Flow absolute | 890 (187) | 892 (188) | 871 (177) | 0.417 |
| MeanASCP flow Indexed | 11.5 (2.04) | 11.5 (2.01) | 11.3 (2.30) | 0.568 |
| Age (y) | 64.3 (12.0) | 64.3 (12.1) | 64.6 (11.2) | 0.853 |
| Female | 167 (34.0%) | 146 (33.4%) | 21 (38.9%) | 0.516 |
| Weight(kg) | 79.0 (17.3) | 79.1 (17.7) | 78.2 (13.8) | 0.672 |
| Height(cm) | 171 (10.2) | 171 (10.2) | 172 (9.79) | 0.668 |
| BSA(mq) | 1.93 (0.25) | 1.93 (0.25) | 1.92 (0.21) | 0.832 |
| BMI | 26.8 (4.82) | 26.9 (4.94) | 26.4 (3.74) | 0.439 |
| EuroSCOREII(%) | 8.27 (5.81) | 7.95 (4.67) | 11.2 (11.7) | 0.069 |
| LVEF(%) | 59.7 (6.74) | 59.6 (6.91) | 60.3 (4.97) | 0.411 |
| Preoperative Renal Failure n(%) | 42 (8.61%) | 37 (8.53%) | 5 (9.26%) | 0.798 |
| Diabetes n(%) | 32 (6.54%) | 27 (6.21%) | 5 (9.26%) | 0.381 |
| Smoking n(%) | 190 (38.8%) | 173 (39.7%) | 17 (31.5%) | 0.309 |
| COPD n(%) | 2 (3.64%) | 2 (4.26%) | 0 (0.00%) | 1.000 |
| TIA n(%) | 490 (100%) | 436 (100%) | 54 (100%) | . |
| Preoperative Stroke n(%) | 491 (100%) | 437 (100%) | 54 (100%) | . |
| Marfan n(%) | 13 (2.66%) | 12 (2.76%) | 1 (1.85%) | 1.000 |
| Loeys Dietz n(%) | 1 (0.20%) | 1 (0.23%) | 0 (0.00%) | 1.000 |
| REDO SURGERY n(%) | 133 (27.2%) | 125 (28.7%) | 8 (14.8%) | 0.045 |
| Urgency: |  |  |  | <0.001 |
| Elective | 205 (41.8%) | 195 (44.6%) | 10 (18.5%) |  |
| Urgency/Emergency | 286 (58.2%) | 242 (55.4%) | 44 (81.5%) |  |
| Type B Dissection n(%) | 29 (5.91%) | 28 (6.41%) | 1 (1.85%) | 0.234 |
| Type A Dissection n(%) | 232 (47.3%) | 191 (43.7%) | 41 (75.9%) | <0.001 |
| Aneurysm n(%) | 181 (36.9%) | 173 (39.6%) | 8 (14.8%) | 0.001 |
| Replacement Extension n(%) |  |  |  | 0.628 |
| Elephant Trunk | 22 (4.48%) | 20 (4.58%) | 2 (3.70%) |  |
| Frozen Elephant Trunk | 179 (36.5%) | 163 (37.3%) | 16 (29.6%) |  |
| Hemiarch | 183 (37.3%) | 162 (37.1%) | 21 (38.9%) |  |
| Other | 5 (1.02%) | 5 (1.14%) | 0 (0.00%) |  |
| Partial/Total Arch | 102 (20.8%) | 87 (19.9%) | 15 (27.8%) |  |
| Cannulation_Type n(%) |  |  |  | 0.467 |
| Arch | 16 (3.26%) | 15 (3.43%) | 1 (1.85%) |  |
| AscendingAorta | 52 (10.6%) | 50 (11.4%) | 2 (3.70%) |  |
| Axillary | 80 (16.3%) | 71 (16.2%) | 9 (16.7%) |  |
| Axillary+Carotid | 1 (0.20%) | 1 (0.23%) | 0 (0.00%) |  |
| BCT | 140 (28.5%) | 126 (28.8%) | 14 (25.9%) |  |
| Carotid | 43 (8.76%) | 38 (8.70%) | 5 (9.26%) |  |
| Femoral | 159 (32.4%) | 136 (31.1%) | 23 (42.6%) |  |
| Concomitant CABG n(%) | 31 (6.31%) | 29 (6.64%) | 2 (3.70%) | 0.560 |
| Concomitant AVR n(%) | 22 (4.48%) | 22 (5.03%) | 0 (0.00%) | 0.154 |
| Concomitant Bentall n(%) | 175 (35.6%) | 150 (34.3%) | 25 (46.3%) | 0.013 |
| CPB Time (min) | 221 (65.4) | 219 (65.6) | 234 (63.0) | 0.112 |
| Aortic clamp (min) | 138 (50.0) | 137 (50.0) | 149 (49.1) | 0.087 |
| Circulatory arrest time (min) | 3.88 (12.8) | 4.04 (13.5) | 2.56 (4.05) | 0.082 |
| Time of ASCP (min) | 74.8 (45.6) | 74.9 (46.1) | 74.2 (41.3) | 0.900 |
| Time of Visceral Ischemia (min) | 40.5 (15.6) | 40.3 (15.8) | 42.3 (13.8) | 0.337 |
| Nasopharingeal Temp (°C) | 25.0 (1.03) | 25.0 (1.06) | 25.0 (0.69) | 0.790 |
| ICU stay (days) | 11.3 (19.2) | 9.63 (17.8) | 24.4 (24.2) | <0.001 |
| Hospital stay (days) | 24.8 (24.0) | 22.7 (22.0) | 41.5 (31.8) | <0.001 |
| Intubation Longer than 72 hours n(%) | 135 (28.0%) | 99 (23.1%) | 36 (66.7%) | <0.001 |

ASCP: antegrade selective cerebral perfusion, AVR: aortic valve replacement; BCT: brachiocephalic trunk; BMI: body mass index; BSA: body surface area; CABG: coronary artery bypass grafting; CPB: cardiopulmonary bypass; COPD: chronic obstructive pulmonary disease; ICU: intensive care unit; LVEF: left ventricular ejection fraction; PND: permanent neurological dysfunction.
