# Supplementary material for: Impact of Antegrade Selective Cerebral Perfusion Flow Ranges on Clinical and Neurological Outcomes in Aortic Arch Surgery
Source: Interdiscip Cardiovasc Thorac Surg. 2026 Jul 15;41(8):ivag200. doi: 10.1093/icvts/ivag200 (PMC13431124; doi:10.1093/icvts/ivag200)
Supplement: ivag200_Supplementary_Data [file ivag200_supplementary_data.zip › TABLE 3 SUPPLEMENTARY.docx]

TABLE 3 SUPPLEMENTARY

|  | Overall  n=492 | Absence of transient neurological deficit  n=393 | Transient neurological deficit n=99 | p.overall |
| --- | --- | --- | --- | --- |
| ASCP_Category_Indexed: |  |  |  | 0.156 |
| High (>15 mL/kg/min) | 26 (5.28%) | 24 (6.11%) | 2 (2.02%) |  |
| Low (<10 mL/kg/min) | 71 (14.4%) | 53 (13.5%) | 18 (18.2%) |  |
| Optimal (10-15 mL/min) | 395 (80.3%) | 316 (80.4%) | 79 (79.8%) |  |
| Mean ASCP Flow absolute | 890 (187) | 894 (191) | 875 (169) | 0.331 |
| MeanASCP flow Indexed | 11.5 (2.04) | 11.5 (2.09) | 11.2 (1.77) | 0.135 |
| Age (y) | 64.3 (12.0) | 64.0 (12.2) | 65.5 (11.3) | 0.253 |
| Female | 167 (33.9%) | 135 (34.4%) | 32 (32.3%) | 0.793 |
| Weight(kg) | 79.0 (17.2) | 79.0 (17.3) | 79.2 (16.9) | 0.916 |
| Height(cm) | 171 (10.2) | 172 (10.1) | 170 (10.6) | 0.251 |
| BSA(mq) | 1.93 (0.25) | 1.93 (0.25) | 1.92 (0.24) | 0.918 |
| BMI | 26.8 (4.82) | 26.7 (4.85) | 27.2 (4.71) | 0.350 |
| EuroSCOREII(%) | 8.26 (5.80) | 8.35 (6.26) | 7.93 (3.56) | 0.398 |
| LVEF(%) | 59.7 (6.74) | 59.9 (6.72) | 59.2 (6.82) | 0.384 |
| Preoperative Renal Failure n(%) | 42 (8.59%) | 30 (7.67%) | 12 (12.2%) | 0.214 |
| Diabetes n(%) | 32 (6.53%) | 24 (6.14%) | 8 (8.08%) | 0.638 |
| Smoking n(%) | 190 (38.7%) | 155 (39.5%) | 35 (35.4%) | 0.516 |
| COPD n(%) | 2 (3.64%) | 2 (4.76%) | 0 (0.00%) | 1.000 |
| TIA n(%) | 491 (100%) | 392 (100%) | 99 (100%) | . |
| Preoperative Stroke n(%) | 492 (100%) | 393 (100%) | 99 (100%) | . |
| Marfan n(%) | 13 (2.65%) | 12 (3.07%) | 1 (1.01%) | 0.481 |
| Loeys Dietz n(%) | 1 (0.20%) | 1 (0.26%) | 0 (0.00%) | 1.000 |
| REDO SURGERY | 134 (27.3%) | 108 (27.6%) | 26 (26.5%) | 0.939 |
| Urgency: |  |  |  | 0.175 |
| Elective | 206 (41.9%) | 171 (43.5%) | 35 (35.4%) |  |
| Urgency/Emergency | 286 (58.1%) | 222 (56.5%) | 64 (64.6%) |  |
| Type B Dissection n(%) | 29 (5.89%) | 24 (6.11%) | 5 (5.05%) | 0.873 |
| Type A Dissection n(%) | 232 (47.2%) | 183 (46.6%) | 49 (49.5%) | 0.682 |
| Aneurysm n(%) | 182 (37.0%) | 149 (37.9%) | 33 (33.3%) | 0.467 |
| Replacement Extension n(%): |  |  |  | 0.337 |
| Elephant Trunk | 22 (4.47%) | 20 (5.09%) | 2 (2.02%) |  |
| Frozen Elephant Trunk | 179 (36.4%) | 143 (36.4%) | 36 (36.4%) |  |
| Hemiarch | 183 (37.2%) | 149 (37.9%) | 34 (34.3%) |  |
| Other | 5 (1.02%) | 3 (0.76%) | 2 (2.02%) |  |
| Partial/Total Arch | 103 (20.9%) | 78 (19.8%) | 25 (25.3%) |  |
| Cannulation Type n(%): |  |  |  | 0.437 |
| Arch | 16 (3.25%) | 15 (3.82%) | 1 (1.01%) |  |
| Ascending Aorta | 52 (10.6%) | 40 (10.2%) | 12 (12.1%) |  |
| Axillary | 80 (16.3%) | 67 (17.0%) | 13 (13.1%) |  |
| Axillary+Carotid | 1 (0.20%) | 1 (0.25%) | 0 (0.00%) |  |
| BCT | 141 (28.7%) | 113 (28.8%) | 28 (28.3%) |  |
| Carotid | 43 (8.74%) | 37 (9.41%) | 6 (6.06%) |  |
| Femoral | 159 (32.3%) | 120 (30.5%) | 39 (39.4%) |  |
| Concomitant CABG n(%) | 31 (6.30%) | 25 (6.36%) | 6 (6.06%) | 1.000 |
| Concomitant AVR n(%) | 22 (4.47%) | 18 (4.58%) | 4 (4.04%) | 1.000 |
| Concomitant Bentall n(%) | 175 (35.6%) | 136 (34.6%) | 39 (39.4%) | 0.530 |
| CPB Time (min) | 221 (65.3) | 221 (66.1) | 222 (62.5) | 0.925 |
| Aortic clamp time (min) | 138 (50.0) | 138 (49.4) | 139 (52.6) | 0.751 |
| Circulatory arrest time (min) | 3.87 (12.8) | 4.35 (14.2) | 1.97 (3.71) | 0.003 |
| Time of ASCP (min) | 74.9 (45.6) | 73.8 (43.4) | 79.3 (53.2) | 0.347 |
| Time of Visceral Ischemia (min) | 40.5 (15.6) | 40.6 (15.3) | 40.4 (16.9) | 0.935 |
| Nasopharingeal Temp (°C) | 25.0 (1.03) | 25.0 (1.07) | 24.9 (0.82) | 0.359 |
| ICU stay (days) | 11.3 (19.2) | 9.36 (17.8) | 19.0 (22.5) | <0.001 |
| Hospital stay (days) | 24.8 (24.0) | 22.4 (22.0) | 34.4 (29.0) | <0.001 |
| Intubation Longer than 72 hours n(%) | 136 (28.1%) | 82 (21.2%) | 54 (55.1%) | <0.001 |
| Complications PND n(%) | 54 (11.0%) | 40 (10.2%) | 14 (14.3%) | 0.326 |
| Stroke | 40 (8.13%) | 29 (7.38%) | 11 (11.1%) | 0.313 |

ASCP: antegrade selective cerebral perfusion, AVR: aortic valve replacement; BCT: brachiocephalic trunk; BMI: body mass index; BSA: body surface area; CABG: coronary artery bypass grafting; CPB: cardiopulmonary bypass; COPD: chronic obstructive pulmonary disease; ICU: intensive care unit; LVEF: left ventricular ejection fraction; PND: permanent neurological dysfunction; TND: transient neurological deficit.

In this table, TND includes: agitation, delirium, transient upper and lower limb deficit, somnolence, aphasia, seizure, transient amnesia, and transient ischemic attack.
